# Effects of arginine vasopressin deficiency on cardiac fibrosis in male Wistar rats

**DOI:** 10.14814/phy2.70748

**Published:** 2026-01-28

**Authors:** A. Limón‐Mendoza, J. Alamilla‐Rasso, S. González‐Nuñez, M. Becerra‐Mendez, S. García‐Álvarez, K. Tinajero‐Vidales, M. Tinajero‐Ruelas, A. Quintanar‐Stephano

**Affiliations:** ^1^ Laboratorio de Neuroinmunoendocrinología. Departamento de Fisiología y Farmacología. Centro de Ciencias Básicas Universidad Autónoma de Aguascalientes Aguascalientes México

**Keywords:** cardiac fibrosis, conivaptan, hypertensive hearth disease, vasopressin

## Abstract

Cardiac fibrosis represents a consequence of hypertensive heart disease and is associated with ventricular dysfunction, arrhythmias, and mortality. Arginine vasopressin (AVP) promotes myofibroblast proliferation and collagen synthesis through V1a receptors. While hepatic studies suggest that AVP deficiency attenuates fibrosis, its cardiac impact remains unclear. This study evaluated the effects of AVP deficiency induced by neurointermediate pituitary lobectomy (NIL) and pharmacological V1a/V2 blockade with conivaptan (CV) in rats with fibrosis from abdominal aortic stenosis (AAC). Wistar rats were divided into seven groups with 10 animals each. Clinical variables and histopathology (H&E, Masson's trichrome, picrosirius red) were assessed. ANOVA, Fisher and Mantel‐Cox tests were applied. The Fibrosis (F) group developed hypertrophy, hypertension, higher arrhythmia risk and increased fibrosis. In contrast, F+NIL and F+CV showed blood pressure and cardiac morphology comparable to controls, reduced arrhythmia risk and significantly less fibrosis. Histologically, F+NIL achieved partial regression, whereas F+CV nearly normalized tissue architecture. In conclusion, AVP deficiency or receptor blockade decreases and reverses AAC‐induced fibrosis, improving hemodynamic, electrical, and structural outcomes. V1a/V2 blockade emerges as a potential therapeutic strategy.

## 1 INTRODUCTION

Cardiovascular diseases represent one of the most leading causes of morbidity and mortality worldwide. Among the most relevant entities are heart failure and hypertensive heart disease, both sharing pathophysiological mechanisms centered on structural myocardial remodeling and the development of cardiac fibrosis.

Heart failure is defined as a clinical syndrome characterized by dyspnea, fatigue, peripheral edema, and orthopnea, secondary to the heart's incapacity to maintain an adequate cardiac output to meet the metabolic demands (Heidenreich, 2024; Khan et al., 2024). Its prevalence in the western countries ranges between 1% and 14%, with a 5 years survival rate below 50%. Due to population aging, the prevalence is expected to increase by up to 46% by 2030 (Heidenreich, 2024).

Hypertensive heart disease represents the structural and functional myocardial alterations induced by chronic pressure overload induced by systemic arterial hypertension, characterized by the progressive development of left ventricular hypertrophy and cardiac fibrosis. In the PARADIGM‐HF trial, up to 70% of patients with chronic heart failure had a background of systemic arterial hypertension; other studies have shown that hypertension is responsible for at least 50% of all global heart failure cases (Slivnick & Lampert, 2019).

The common pathological mechanism that links both heart failure and hypertensive heart disease is myocardial fibrosis formation, defined as expansion of the interstitium due to pathological accumulation of extracellular matrix proteins, mainly type I and III collagen caused by persistent myofibroblast activation. At the molecular level, fibrosis is induced by the activation and differentiation of the fibroblast into active myofibroblast; this process is regulated by angiotensin II (AT1R), aldosterone (MAPK pathway), transforming growth factor beta (TGF‐β), mechanical distention through mechanosensitive channels, and proinflammatory cytokines (IL‐6, IL‐11, and endothelin‐1) (Frangogiannis, 2021).

In hypertensive heart disease, sustained ventricular afterload increases left ventricular wall stress, triggering compensatory concentric hypertrophy. This process activates mechanosensitive channels that stimulate collagen synthesis through MAPK, PI3K, and calcineurin pathways leading to extracellular matrix deposition and fibrosis development (Slivnick & Lampert, 2019). Chronic activation of the renin‐angiotensin‐aldosterone system (RAAC) and oxidative stress enhances this process (Slivnick & Lampert, 2019). In congestive heart failure, fibrosis arises from activation and proliferation of fibroblasts through mechanoreceptors activated due to chronic overstretch, along with stimulation of TGF‐β and AT1R pathways (Moore‐Morris et al., 2015).

Histopathologically, cardiac fibrosis is classified into three main patterns: replacement, interstitial, and perivascular fibrosis. Replacement fibrosis involves substitution of necrotic cardiomyocytes with collagenous scar tissue, as observed after acute myocardial infarction; this process preserves structural integrity but reduces contractility, compromising global ventricular function (Frangogiannis, 2021). Interstitial fibrosis is characterized by diffuse expansion of the interstitium without cell loss, is the result from chronic inflammatory or mechanical stimuli and leads to ventricular dysfunction (Ieronimakis et al., 2013). Perivascular fibrosis is defined by the accumulation of collagen in the adventitia of coronary arteries, is caused by chronic overload stimuli; the consequences of this process are the reduction in myocardial perfusion that leads to stable angina or acute coronary syndromes (Fan et al., 2007). Both hypertensive heart disease and congestive heart failure predominantly induce interstitial and perivascular fibrosis (Frangogiannis, 2021).

**FIGURE 1:**
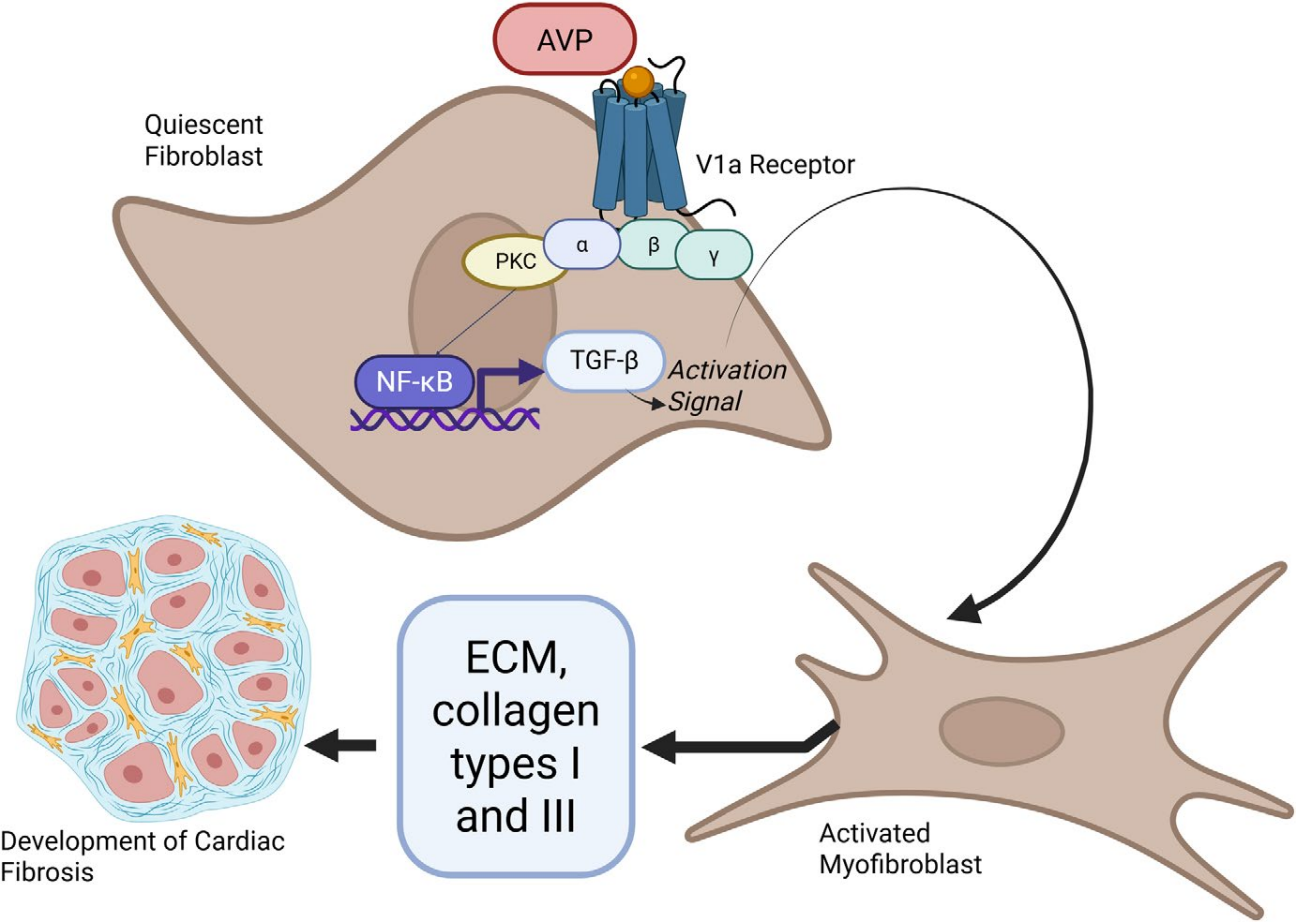
Schematic illustration of V1a receptor–mediated actions on cardiac fibroblasts. This schematic illustrates the proposed actions of the V1a receptor on cardiac fibroblasts based on previously reported mechanisms. AVP binds to the V1a receptor, a Gq‐coupled G protein receptor, whose α‐subunit activates protein kinase C (PKC). PKC stimulates the nuclear factor kappa B (NF‐κB) signaling pathway, promoting the transcription and release of transforming growth factor beta (TGF‐β). This factor acts as an activation signal, inducing the proliferation and differentiation of quiescent fibroblasts into active myofibroblasts, which synthesize type I and III collagen and other extracellular matrix (ECM) proteins. The progressive accumulation of these components leads to the development of cardiac fibrosis.

Arginine vasopressin (AVP) has been identified as an important inducer of cardiac fibrosis. Its effect is primarily mediated through a direct mechanism, mediated by the AVP binding to the V1a receptor, which activates the protein kinase C (PKC), which in turn stimulates the NF‐kB signaling pathway, promoting TGF‐β expression, that directly activates myofibroblast and enhances collagen synthesis (Fan et al., 2007; Zeisberg et al., 2007) (Figure 1).

Several animal models have been developed to induce cardiac fibrosis; abdominal aortic stenosis (EA) is one of the most used (Houser et al., 2012). This surgical procedure consists of reducing the abdominal aortic diameter with a ligature placed at the level of the renal arteries, leading to increased left ventricular ejection resistance and, consequently, sustained afterload elevation. Initially, this condition triggers compensatory concentric hypertrophy; however, over time, this adaptive response transforms into pathological remodeling culminating in myocardial fibrosis (Ku et al., 2016). The proposed molecular mechanisms are mediated by the activation of mechano‐transduction pathways in cardiomyocytes and fibroblasts, as well as RAAC activation secondary to reduced renal blood flow. Both converge in myofibroblast stimulation, the key effector cells in collagen synthesis and deposition. The fibrosis generated in this model is predominantly interstitial and perivascular, closely reproducing the histological pattern observed in hypertensive heart disease. A key feature of AAC is that structural changes develop chronically: during the early weeks, the myocardium maintains contractile capacity to compensate for pressure overload; however, fibrotic alterations become evident after the eighth week (Ajith Kumar et al., 2014). This model offers significant advantages, for example, it does not require thoracotomy, reducing operative mortality, and mainly produces progressive and stable pressure overload, similar to the clinical course of hypertensive cardiopathy in humans (Ku et al., 2016).

On another hand, experimental models of liver fibrosis have shown that AVP deficiency, either surgically induced by neurointermediate pituitary lobectomy (NIL), a procedure involving neurohypophyseal resection, or pharmacologically blockade of its receptors (V1a/V2) with conivaptan (CV), reduces progression and even reverses hepatic fibrosis induced by portocaval anastomosis (Muñoz‐Ortega et al., 2021; Navarro‐Gonzalez et al., 2023; Quintanar‐Stephano et al., 2017). The proposed molecular mechanisms indicate that AVP deficiency promotes a fibrolytic microenvironment, characterized by increased expression of metalloproteinases (MMP‐13) and reduced expression of tissue inhibitors of metalloproteinases (TIMP‐2), facilitating extracellular matrix degradation and reducing total fibrosis percentage (Muñoz‐Ortega et al., 2021; Navarro‐Gonzalez et al., 2023; Quintanar‐Stephano et al., 2017). This effect is attributed to the lack of V1a/V2 receptor activation in liver stellate cells, the main mediators of hepatic fibrogenesis (Muñoz‐Ortega et al., 2021).

In the cardiovascular field, AVP—through its V1a receptor—has been shown to stimulate proliferation and differentiation of cardiac myofibroblasts, promoting collagen deposition and fibrotic remodeling (Fan et al., 2007). Based on this cross‐organ evidence, we hypothesize that AVP deficiency induced by NIL or pharmacological V1a/V2 receptor blockade with CV may attenuate cardiac myofibroblast activation, thereby reducing the progression or even inducing regression of myocardial fibrosis. To demonstrate this hypothesis, we evaluated the effects of both interventions on clinical variables (noninvasive tail‐cuff blood pressure, invasive carotid pressure, resting electrocardiogram (ECG), exercise test, survival, and cardiac morphometry) and histopathological parameters (Hematoxylin–Eosin, Masson's trichrome staining and picrosirius red staining under non and with polarized light).

## 2 MATERIALS AND METHODS

### 2.1 Animals and experimental protocol

Male Wistar rats (220 g of corporal weight and 6 weeks old) were obtained from the animal facility of the Universidad Autónoma de Aguascalientes. Animals were maintained on a 12:12 h light–dark cycle (lights on at 07:00) in a temperature‐ and humidity‐controlled environment (22 ± 1°C). They received LabDiet 5001 nutriblocks and water ad libitum. All procedures conformed to the institutional guidelines of the Bioethics Committee of the Universidad Autónoma de Aguascalientes and to Mexican regulations in strict accordance with the Norma Oficial Mexicana “Especificaciones técnicas para la producción, cuidado y uso de los animales de laboratorio” (NOM‐062‐ZOO‐1999). The experimental protocol was approved by the Institutional Bioethics Committee of the Universidad Autónoma de Aguascalientes (approval ID CEADI‐UAA/02/2024).

Rats were allocated into seven experimental groups: Intact Control (IC), Neurointermediate pituitary lobectomy (NIL), Conivaptan (CV), Dimethyl sulfoxide (DMSO, vehicle for CV), Fibrosis (F), F+NIL, and F+CV. Each group initially included 10 animals (*n* = 10), 70 animals in total. This sample size is traditionally accepted in experimental cardiovascular and histopathological studies as sufficient to ensure biological reproducibility while minimizing animal use in accordance with ethical principles. The experiment lasted 12 weeks. Animals in groups F, F+NIL, and F+CV underwent abdominal aortic stenosis surgery at week 0. Therapeutic interventions began at week 6: CV and DMSO administration started in their respective groups, whereas NIL surgeries were performed in the NIL and F+NIL groups. Euthanasia was performed at week 12.

**FIGURE 2:**
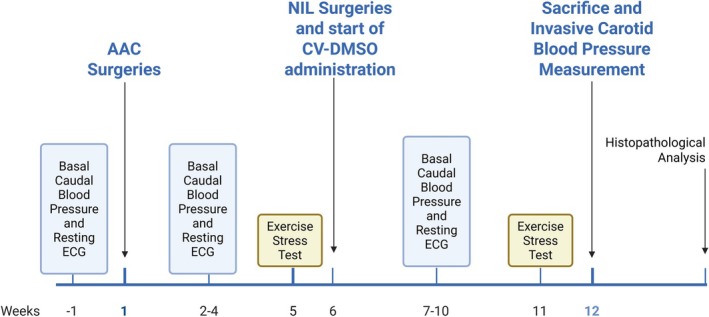
Experimental timeline. One week before the start of the experiment, baseline measurements of caudal cuff‐tail blood pressure, resting ECG were obtained. At week 1, abdominal aortic stenosis (AAC) surgery was performed on the F, F+NIL, and F+CV groups. During weeks 2–4, serial weekly measurements of caudal blood pressure, resting ECG, and were carried out. At week 5, the first forced‐swim exercise test was conducted. At week 6, NIL surgeries were performed in the NIL and F+NIL groups, while DMSO and CV treatments were administrated in their respective groups (CV, DMSO, and F+CV). During weeks 7–10, serial weekly measurements of caudal blood pressure, resting ECG were repeated. At week 11, the final forced‐swim exercise test was performed. At week 12, invasive carotid blood pressure was measured, followed by euthanasia, cardiac morphometric measurements, and collection of histopathological samples for H‐E, Picrosirius Red, and Masson's trichrome staining.

Clinical variables (body weight, tail‐cuff blood pressure, resting ECG, and exercise test) were recorded serially throughout the study. Invasive carotid blood pressure was measured under surgical anesthesia prior to euthanasia. The detailed experimental timeline is shown in Figure 2.

### 2.2 Neurointermediate pituitary lobectomy

The NIL procedure was performed according to Quintanar‐Stephano et al. (Navarro‐Gonzalez et al., 2023; Quintanar‐Stephano et al., 2005). Surgeries were carried out under a Zeiss OPMI‐19 FC stereoscopic microscope at 6× magnification. Rats were anesthetized with isoflurane (Sofloran Vet. PISA, Q‐7833‐222) at 3% for induction and 1.5% for maintenance. Average operative time was 6 min per animal. Postoperatively, animals were transferred to a recovery chamber and received a single dose of 11,000 IU/kg of Penicillin G Benzathine–Penicillin G Procaine–Penicillin G Sodium–Dihydrostreptomycin–Dexamethasone–Dipyrone (500,000 IU/375,000 IU/125,000 IU/375 mg/10 mg/250 mg – 5 mL; DEXA‐Estreptovet, AGROVET Laboratory, Q‐0024‐089) used as postoperative analgesic, anti‐inflammatory, and antibiotic prophylaxis. Full recovery was achieved 60 min after surgery.

### 2.3 Abdominal aortic stenosis

AAC was performed based on Hui‐Chun Ku et al. Technique (Ku et al., 2016), with minor modifications. The main change was placing the abdominal aortic ligature immediately below the origin of the renal arteries. This modification, standardized in our laboratory, yielded equivalent efficacy for inducing cardiac fibrosis while being technically simpler and reducing short‐ and mid‐term operative morbimortality. Rats were anesthetized with isoflurane (Sofloran Vet., PISA, Q‐7833‐222) at 3% for induction and 1.5% for maintenance. Average operative time was 15 min per animal. Postoperatively, animals were moved to a recovery chamber and received a single dose of 11,000 IU/kg of Penicillin G Benzathine–Penicillin G Procaine–Penicillin G Sodium–Dihydrostreptomycin–Dexamethasone–Dipyrone (500,000 IU/375,000 IU/125,000 IU/375 mg/10 mg/250 mg – 5 mL; DEXA‐Estreptovet, AGROVET Laboratorio, Q‐0024‐089) as analgesic, anti‐inflammatory, and antibiotic prophylaxis. Complete recovery was observed 120 min after surgery.

### 2.4 Conivaptan

The CV dose (BioChemPartner, China; catalog #BCP284426) was 1 mg/kg, following Quintanar‐Stephano et al. standardization (Navarro‐Gonzalez et al., 2023). A stock solution was prepared by dissolving 30 mg of CV in 1 mL dimethyl sulfoxide (DMSO), the vehicle for CV (Sigma‐Aldrich, Germany; catalog #276855). From this stock, 100 μL were diluted in 900 μL of sterile injectable water (PISA, Mexico; catalog #75592). 100 μL of this working solution (equivalent to 0.3 mg per application) were administered every 24 h for 6 weeks intramuscularly, starting at week 6 in the CV and F + CV groups. The DMSO group received vehicle only (DMSO diluted in sterile water at the same proportion) in the same volume and frequency as the CV‐treated group.

### 2.5 Measurement of clinical variables

#### 2.5.1 Body weight

All experimental groups were weighed weekly to monitor body weight and progression throughout the study. Deaths were recorded to calculate survival rate at the end of the protocol.

#### 2.5.2 Noninvasive caudal blood pressure (tail‐cuff)

Systolic blood pressure of the caudal artery was measured noninvasively using the tail‐cuff method with a TSD104A pressure transducer and a TSD200 pulse transducer, both connected to the MP150 data acquisition system (BIOPAC Systems Inc., USA). Recordings were processed with AcqKnowledge v4.1 (BIOPAC Systems Inc.) to obtain average systolic pressure values for each experimental session.

#### 2.5.3 Resting electrocardiogram (ECG)

For resting ECG recording, animals were anesthetized with 1.5% isoflurane for 5 min. Lead II was recorded using an ECG100C amplifier connected to the MP150 physiological recording system (BIOPAC Systems Inc.). Reference ECG parameters were established according to Konopelski and Ufnal (2016) The relative risk of cardiac arrhythmias was determined considering the presence of ST‐segment elevation or depression, peaked Q waves, and atrioventricular or right/left bundle branch blocks.

#### 2.5.4 Exercise stress test

The exercise test protocol was adapted from Oláh et al. (2015). Rats were placed individually in a cylindrical tank (30 cm diameter × 50 cm height) filled with 20 L of water at 37.5°C. Forced swimming was induced for 10 min, and “active swimming time”—defined as the period with simultaneous movement of all four limbs—was recorded as the exercise tolerance time. At the end of the 10 min, rats were carefully dried, and then immediately anesthetized with 1.5% isoflurane for 5 min while a post‐exercise ECG was obtained to assess for exercise‐induced arrhythmias.

### 2.6 Invasive carotid blood pressure, euthanasia, and cardiac morphometry

#### 2.6.1 Invasive carotid blood pressure

For mean carotid arterial pressure measurement, rats were anesthetized with isoflurane (Sofloran Vet., PISA, Q‐7833‐222) at 3% for induction and 1.5% for maintenance. After achieving surgical anesthesia, animals were placed supine with limbs secured to the surgical table. The trachea was cannulated to maintain airway patency; the anterior neck area was shaved and disinfected; a 2 cm longitudinal cervical incision was made. Under a stereoscopic microscope, the carotid artery was dissected and isolated, then cannulated with a heparinized catheter. The catheter was secured and connected to a TSD‐104A pressure transducer coupled to the MP150 physiological acquisition system (BIOPAC Systems Inc.), and measurements were recorded using AcqKnowledge v4.1 software. Mean arterial pressure (MAP) was calculated from the average of three consecutive measurements in the same animal.

#### 2.6.2 Euthanasia

Following carotid pressure measurement and under deep surgical anesthesia, thoracic and abdominal cavities were opened to induce the animal's death, and then vascular perfusion was performed with 50 mL of 0.9% saline, followed by 50 mL of 10% neutral buffered formalin using an infusion pump at a constant rate of 1 mL/min. After fixation, hearts were excised for cardiac morphometric assessments.

#### 2.6.3 Cardiac morphometry

Excised hearts were cut by a mid‐transversal incision, and microphotographs were obtained using a stereoscopic microscope at 6× magnification. Images were processed with ImageJ v1.54g. After scale calibration, left ventricular wall thickness was measured in millimeters across all groups. Hearts were then weighed on an analytical balance and expressed as mg per 100 g body weight. Three measurements per heart were obtained in each rat, and the mean value was used for morphometric analysis.

#### 2.6.4 Cardiac histopathology

Cardiac samples were preserved in 10% neutral buffered formalin and processed for histological slides and stained with Hematoxylin–eosin (H‐E), picrosirius red (Sigma‐Aldrych, Catalog #365548), and Masson's trichrome stain (Sigma‐Aldrych, Catalog #HT15). For the anatomopathological assessment, 10–15 fields per animal were analyzed in each experimental group at 10× magnification with an optic microscope (Nikon Optiphot‐2). Images were analyzed with ImageJ v1.54g software to determine the percentage of fibrosis from Masson's trichrome and picrosirius red staining with and without polarized light. In Masson's trichrome, fibrotic areas were identified by blue coloration, while in picrosirius red, collagen fibers showed intense red coloration. Based on these histological features, the percentage of myocardial fibrosis was quantified for each group (Lattouf et al., 2014; Suvik & Effendy, 2012).

### 2.7 Statistical analysis

Results are expressed as mean ± standard deviation (SD). Statistical analyses included ANOVA, Student's *t*‐test, Mantel–Cox test for survival rate, and Fisher's exact test to assess the relative risk of arrhythmias. Differences were considered statistically significant at *p* < 0.05. All analyses were performed using GraphPad Prism v8.0 software.

## 3 RESULTS

**FIGURE 3:**
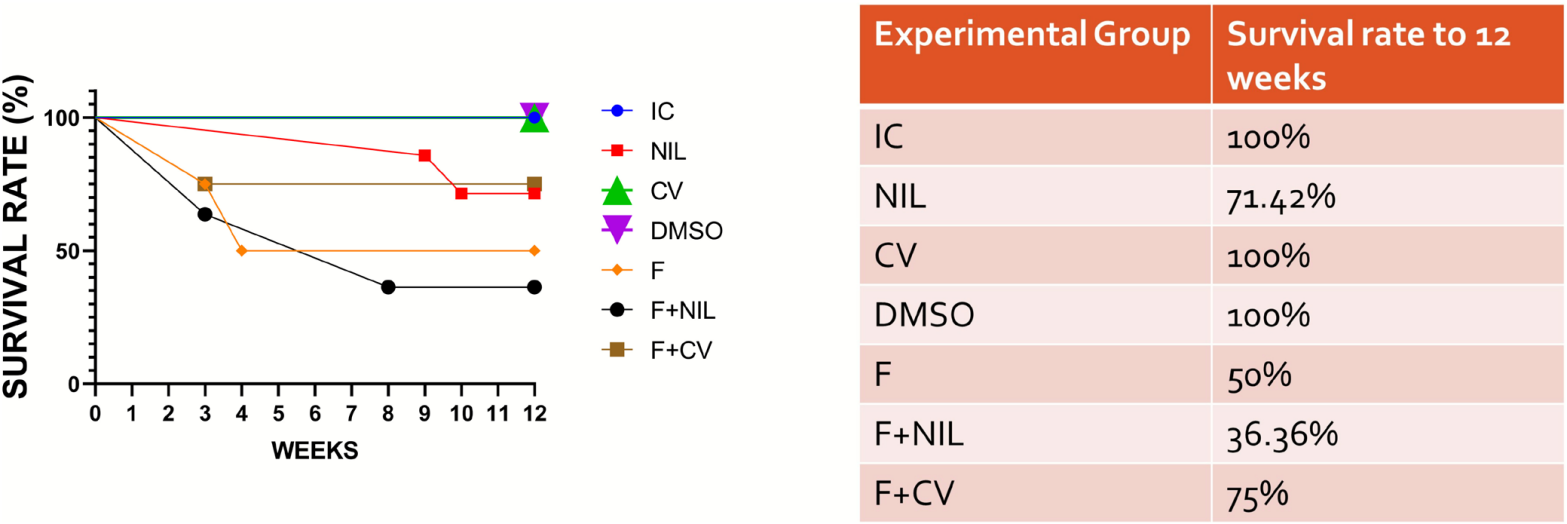
Survival rate. Kaplan–Meier survival curve obtained over the 12‐week experimental period. The F group showed a 50% survival rate, whereas conivaptan treatment (F+CV) improved survival to 75%. In contrast, F+NIL reduced survival to 36.36%, with the highest mortality occurring immediately after surgery. The NIL group also exhibited a decrease in survival to 71.42% following the procedure. Number of animals per group: IC (*n* = 10), NIL (*n* = 7), CV (*n* = 10), DMSO (*n* = 10), F (*n* = 5), F+NIL (*n* = 5), and F+CV (*n* = 8).

The survival curve is shown in Figure 3. The F group showed a 50% survival rate at 12 weeks. CV improved the survival rate to 75% at the F+CV, whereas NIL reduced the survival rate to 36.36% at the F+NIL group; this increment in mortality occurred immediately after surgery; a decrease in the survival rate at the postoperative period was also evident in the NIL group. Rats receiving NIL or CV interventions also exhibited features of diabetes insipidus, which indirectly suggest reduced circulating AVP levels based on clinical findings; however, serum AVP concentrations were not measured.

**FIGURE 4:**
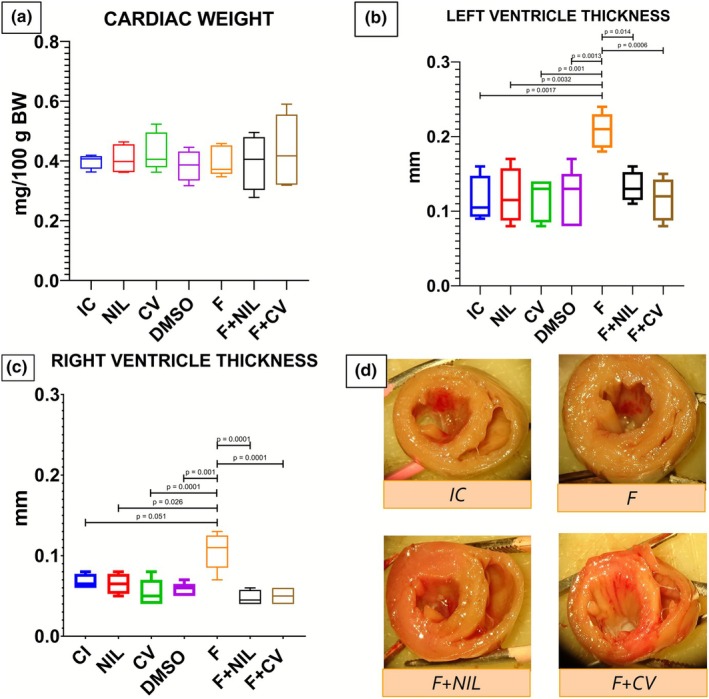
Cardiac morphometry. (a) Total heart weight expressed as mg per 100 g of body weight. No significant differences were observed among experimental groups. (b) Left ventricular wall thickness and (c) right ventricular wall thickness, expressed in millimeters. The F group showed a significant increase in ventricular wall thickness compared with IC, NIL, CV, DMSO, and the treated groups (F+NIL and F+CV) (*p* < 0.05). The F+NIL and F+CV groups showed no evidence of significant hypertrophy. (d) Representative macroscopic transversal heart sections from IC, F, F+CV, and F+NIL groups, illustrating increased ventricular wall thickness in F and its partial reduction in treated groups. Intergroup differences were analyzed using one‐way ANOVA followed by Student's *t*‐test for multiple comparisons. Number of animals per group: IC (*n* = 10), NIL (*n* = 7), CV (*n* = 10), DMSO (*n* = 10), F (*n* = 5), F+NIL (*n* = 5), and F+CV (*n* = 8).

Cardiac morphometry is described in Figure 4. Total heart weight showed no significant differences among experimental groups. However, left and right ventricular wall thickness increased significantly in the F group, suggesting hypertrophy secondary to AAC. In contrast, F+NIL and F+CV did not show hypertrophy, maintaining values similar to controls.

**FIGURE 5:**
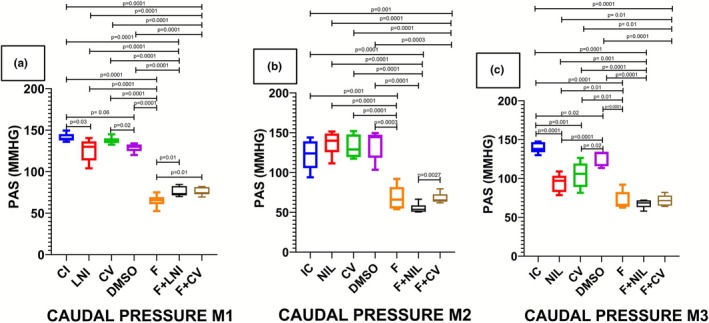
Caudal systolic arterial blood pressure. Three serial measurements were performed throughout the experiment. (a) First measurement, taken 1 week after abdominal aortic stenosis, showed a significant decrease in systolic blood pressure in F, F+NIL, and F+CV compared with the other groups as expected. (b) Second measurement (measured at week 5) maintained the same decreasing pattern in these groups. (c) Third measurement, performed 3 weeks after NIL surgery and initiation of CV administration, revealed that—in addition to F, F+NIL, and F+CV—the NIL and CV groups also exhibited a significant reduction in systolic blood pressure. Results are expressed as mean ± standard deviation (SD); intergroup differences were analyzed using one‐way ANOVA followed by Student's *t*‐test (*p* < 0.05). Number of animals per group; M1: IC (*n* = 10), NIL (*n* = 10), CV (*n* = 10), DMSO (*n* = 10), F (*n* = 10), F+NIL (*n* = 10), F+CV (*n* = 10); M2: IC (*n* = 10), NIL (*n* = 10), CV (*n* = 10), DMSO (*n* = 10), F (*n* = 5), F+NIL (*n* = 7), F+CV (*n* = 8); M3: IC (*n* = 10), NIL (*n* = 7), CV (*n* = 10), DMSO (*n* = 10), F (*n* = 5), F+NIL (*n* = 5), F+CV (*n* = 8).

Tail‐cuff systolic blood pressure is presented in Figure 5. Three serial measurements were obtained during the study. The first measurement, taken 1 week after EA surgery, showed a significant decrease in systolic pressure in F, F+NIL, and F+CV as compared with the remaining groups. The second measurement (measured at week 5) preserved the same decreasing pattern in these groups. In the third measurement—performed 3 weeks after NIL surgery and initiation of CV administration—a significant decrease in systolic pressure was observed in NIL and CV groups in addition to the F, F+NIL, and F+CV animals.

**FIGURE 6:**
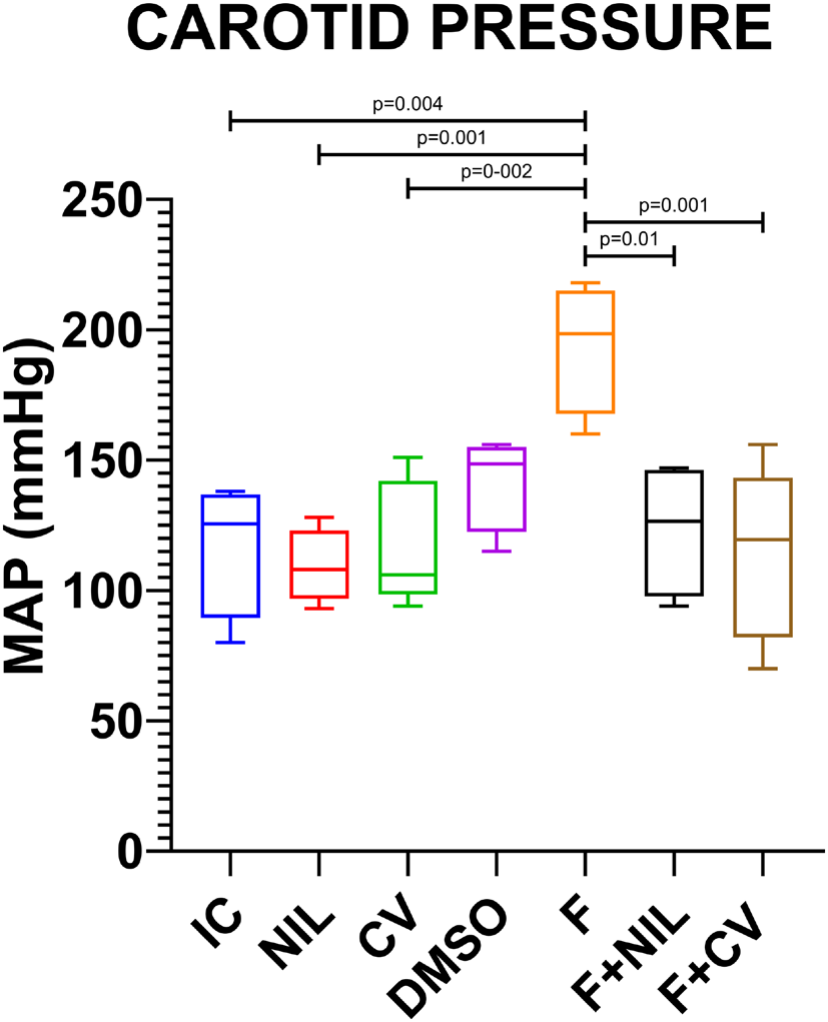
Mean carotid arterial pressure (MAP). A significant increase in MAP was observed in the F group, consistent with the hypertension model induced by abdominal aortic stenosis. In contrast, the F+NIL and F+CV groups showed values within normal physiological ranges, while the NIL and CV groups exhibited no significant differences compared with the control group. Results are expressed as mean ± SD; intergroup comparisons were performed using one‐way ANOVA followed by Student's *t*‐test (p < 0.05). Number of animals per group: IC (*n* = 10), NIL (*n* = 7), CV (*n* = 10), DMSO (*n* = 10), F (*n* = 5), F+NIL (*n* = 5), and F+CV (*n* = 8).

Invasive carotid mean arterial pressure (MAP) is shown in Figure 6. At the end of the experiment, MAP was significantly increased in the F group, consistent with hypertension induced by aortic stenosis. In contrast, F+NIL and F+CV displayed values within normal physiological ranges, and NIL and CV did not differ significantly from controls.

Relative risk (RR) of arrhythmias at rest for groups subjected to AAC (F, F+NIL, and F+CV) is described in Table 1. Arrhythmias were defined as the presence of ST‐segment elevation or depression, peaked Q waves, and atrioventricular or right/left bundle branch blocks. Four weeks after intervention, all groups showed an increased RR compared with week 0. By week 9 (3 weeks after starting CV and after NIL surgery), a marked reduction in RR was observed in the treated groups: from 4.501 to 1.385 in F+NIL, and from 4.909 to 2.929 in F+CV. Conversely, the F group showed a progressive increase in risk, from 2.077 to 5.401.

**TABLE 1:** Relative risk (RR) of developing cardiac arrhythmias on resting ECG.

Experimental group	Weeks^a^	RR	95% IC	*p* Value
IC	Four	Reference		
	Nine			
F	Four	2.077	0.7218 to 11.52	0.5179
	Nine	5.401	1.507 to 30.17	**0.0076**
F+NIL	Four	4.501	1.193 to 25.42	**0.0430**
	Nine	1.385	0.5917 to 7.497	0.9999
F+CV	Four	4.909	1.329 to 27.59	**0.0345**
	Nine	2.929	0.6600 to 10.74	0.5368

*Note*: The table shows the RR, 95% confidence interval (CI 95%), and corresponding *p* values for each experimental group at weeks 4 and 9, compared with week 0. Comparisons were performed using Fisher's exact test, and differences were considered statistically significant when *p* < 0.05. Bold values indicate statiscally significant differences (*p* < 0.05).

^a^ The week comparison was made between week 4 or 9 versus week 0.

**FIGURE 7:**
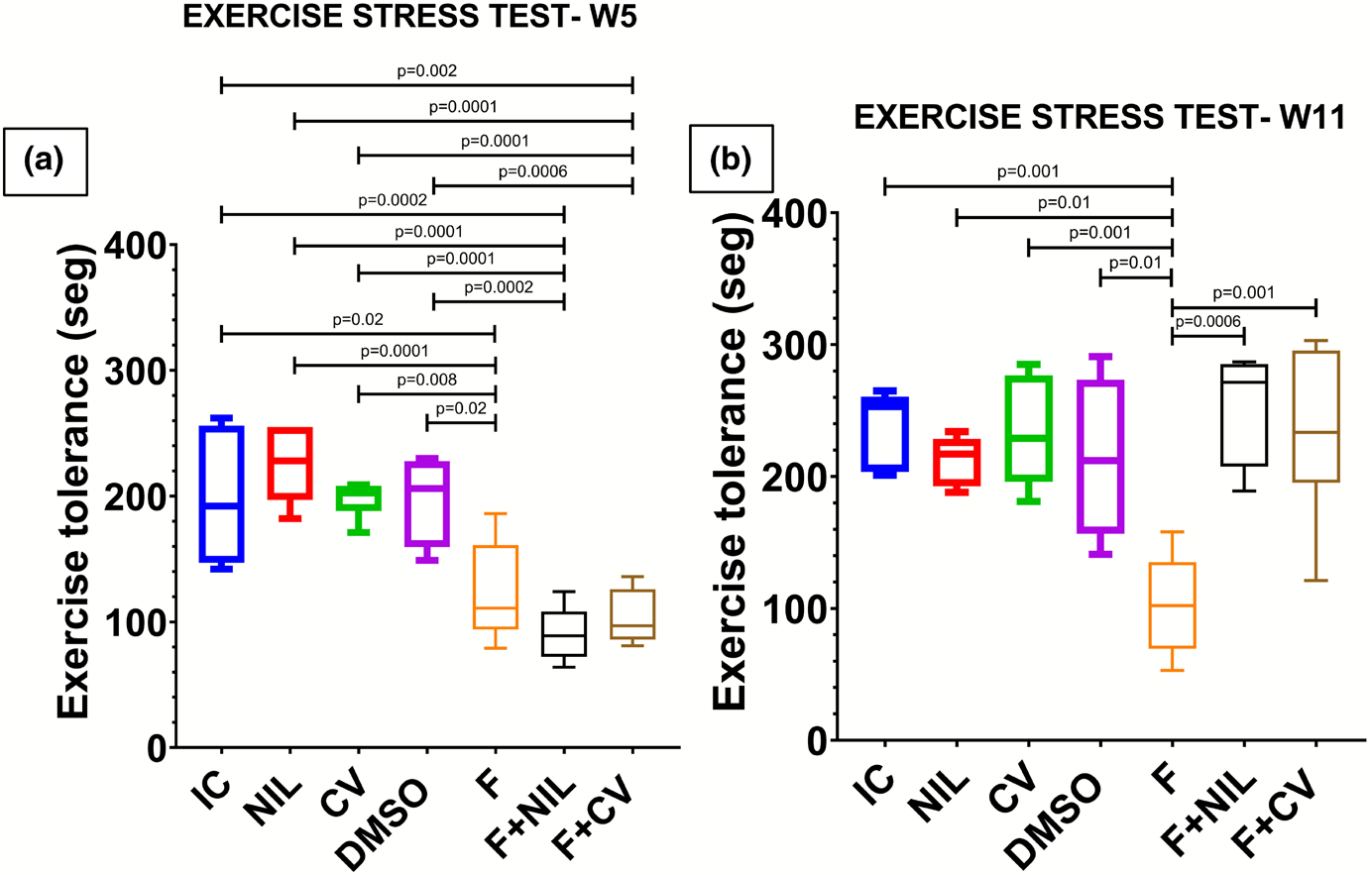
Exercise tolerance in the forced‐swim test. Exercise tolerance time was determined using the forced‐swim test at weeks 5 and 11 of the experiment. (a) At week 5, groups subjected to abdominal aortic stenosis (F, F+NIL, F+CV) showed a significant reduction in active swimming time compared with the other groups. (b) At week 11, the F group maintained low exercise tolerance, whereas the F+NIL and F+CV groups exhibited a significant recovery of exercise capacity, reaching values comparable to the control group. Results are expressed as mean ± SD; intergroup differences were analyzed using one‐way ANOVA followed by Student's *t*‐test (*p* < 0.05). Number of animals per group; Week 5: IC (*n* = 10), NIL (*n* = 10), CV (*n* = 10), DMSO (*n* = 10), F (*n* = 10), F+NIL (*n* = 10), F+CV (*n* = 10); Week 11: IC (*n* = 10), NIL (*n* = 7), CV (*n* = 10), DMSO (*n* = 10), F (*n* = 5), F+NIL (*n* = 5), F+CV (*n* = 8.

Two main parameters were obtained from the forced‐swim test. First of all, exercise tolerance time (Figure 7), at week 5, F, F+NIL, and F+CV showed a significant decrease in exercise tolerance compared with the other groups. By week 11, the F group maintained low tolerance, whereas F+NIL and F+CV exhibited a significant recovery, reaching values comparable to controls. RR of post‐exercise arrhythmias (Table 2): Comparing week 11 with week 5 (after 5 weeks of treatment), F had an RR of 1.5556, while F+NIL and F+CV showed 0.4167 and 0.3571, respectively.

**TABLE 2:** Relative risk (RR) of developing cardiac arrhythmias during the exercise test (post‐exercise ECG).

Experimental group	Weeks	RR	95% IC	*p* Value
IC	Five vs. Eleven	Reference		
F	Five vs. Eleven	1.5556	0.406 to 4.846	**0.036**
F+NIL	Five vs. Eleven	0.4167	0.115 to 1.266	0.2424
F+CV	Five vs. Eleven	0.3571	0.098 to 1.126	0.2424

*Note*: The table presents the relative risk (RR), 95% confidence interval (CI 95%), and *p* value, comparing weeks 5 and 11 of the experiment. Results show that the F group exhibited a significant increase in RR, whereas the treated groups (F+NIL and F+CV) displayed a reduced risk compared with the F group, suggesting a protective effect of AVP deficiency or blockade against exercise‐induced arrhythmias. Comparisons were performed using Fisher's exact test, and differences were considered statistically significant when *p* < 0.05. Bold values indicate statiscally significant differences (*p* < 0.05).

**FIGURE 8:**
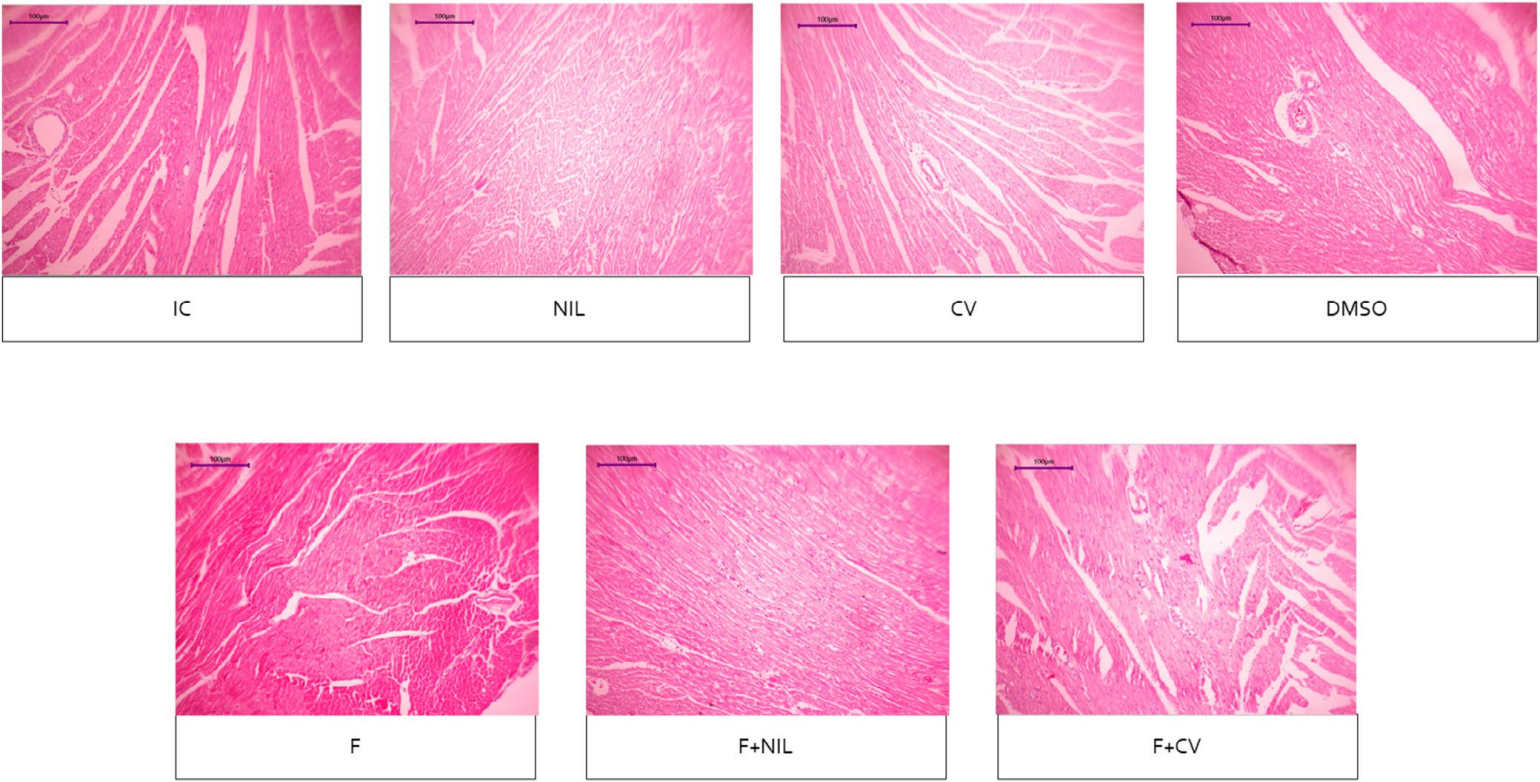
Cardiac histopathological slides under Hematoxylin–Eosin (H‐E) staining, 10× magnification. The IC, NIL, CV, and DMSO groups show a preserved myocardial architecture, with aligned cardiomyocytes, centrally located nuclei, and minimal interstitial expansion. In the F group, there is an evident loss of tissue organization, cardiomyocyte hypertrophy, widened intercellular spaces, and increased spindle‐shaped nuclei consistent with activated fibroblasts, accompanied by a densely expanded interstitium. In contrast, the F + NIL and F + CV groups display improved parenchymal organization, reduced interstitial space, and more uniform cardiomyocyte morphology—particularly in F + CV, where near‐complete preservation of histological integrity and absence of inflammatory infiltrate are observed, suggesting reduced hypertrophy and attenuation of structural remodeling. Scale bar = 100 μm. Number of animals per group: IC (*n* = 10), NIL (*n* = 7), CV (*n* = 10), DMSO (*n* = 10), F (*n* = 5), F+NIL (*n* = 5), and F+CV (*n* = 8).

**FIGURE 9:**
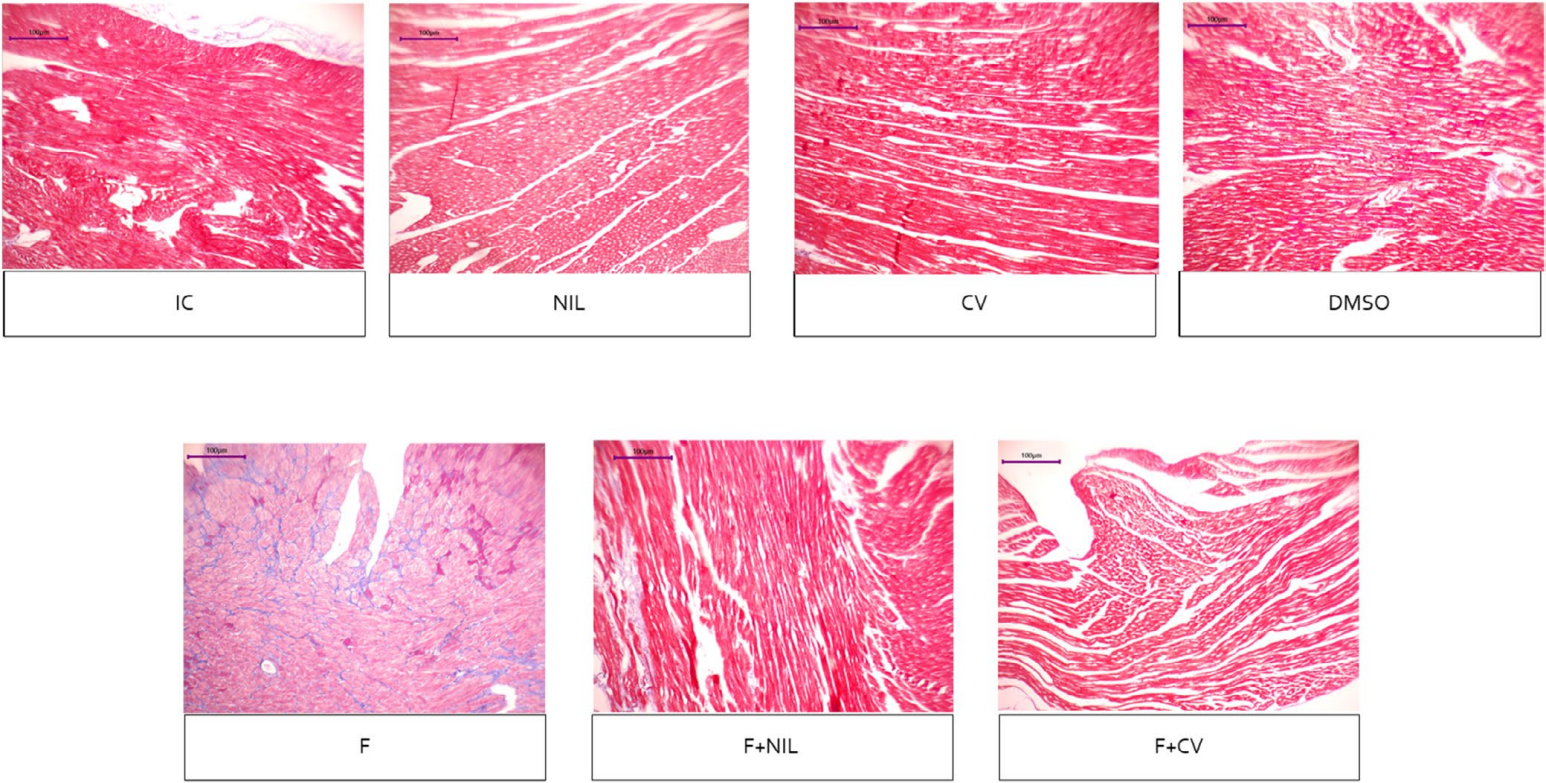
Cardiac histopathological slides under Masson's trichrome staining, 10× magnification. In the IC, NIL, CV, and DMSO groups, the myocardium appears normal, showing minimal interstitial and perivascular collagen, stained as light blue fibers. The F group exhibits extensive deposition of collagen, evidenced by intensely blue‐stained areas occupying both interstitial and perivascular spaces, associated with disorganization of muscle fascicles. In the F+NIL group, a marked reduction in fibrotic areas is observed, with thin, well‐delimited collagen fibers and partial preservation of myocardial architecture. In the F+CV group, staining reveals an almost normal structural pattern, characterized by scattered thin collagen strands, absence of diffuse fibrosis, and preserved cardiac fascicles, indicating a pronounced regression of the fibrotic process. Scale bar = 100 μm. Number of animals per group: IC (*n* = 10), NIL (*n* = 7), CV (*n* = 10), DMSO (*n* = 10), F (*n* = 5), F+NIL (*n* = 5), and F+CV (*n* = 8).

**FIGURE 10:**
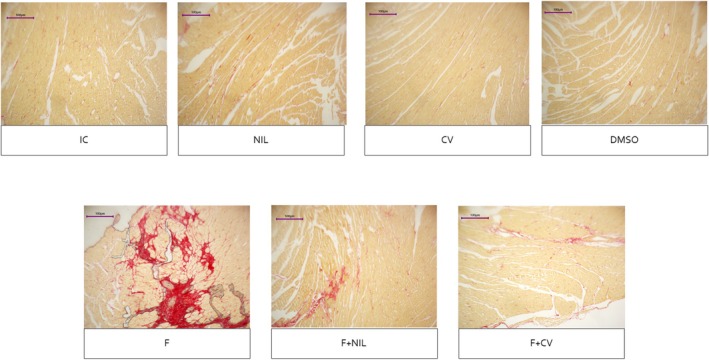
Cardiac histopathological slides under Picrosirius Red staining, 10× magnification. The IC, NIL, CV, and DMSO groups display a normal extracellular matrix pattern, with thin collagen fibers sparsely distributed within the endomysium and around blood vessels. In the F group, staining reveals a massive collagen deposition with intense bright red coloration, indicative of severe interstitial and perivascular fibrosis. The F+NIL group shows an evident reduction in collagen fibers, with a regular and well‐defined distribution, reflecting partial regression of the fibrotic process. The F+CV group exhibits an almost normal pattern, characterized by minimal red staining and preserved myocardial architecture. Scale bar = 100 μm. Number of animals per group: IC (*n* = 10), NIL (*n* = 7), CV (*n* = 10), DMSO (*n* = 10), F (*n* = 5), F+NIL (*n* = 5), and F+CV (*n* = 8).

**FIGURE 11:**
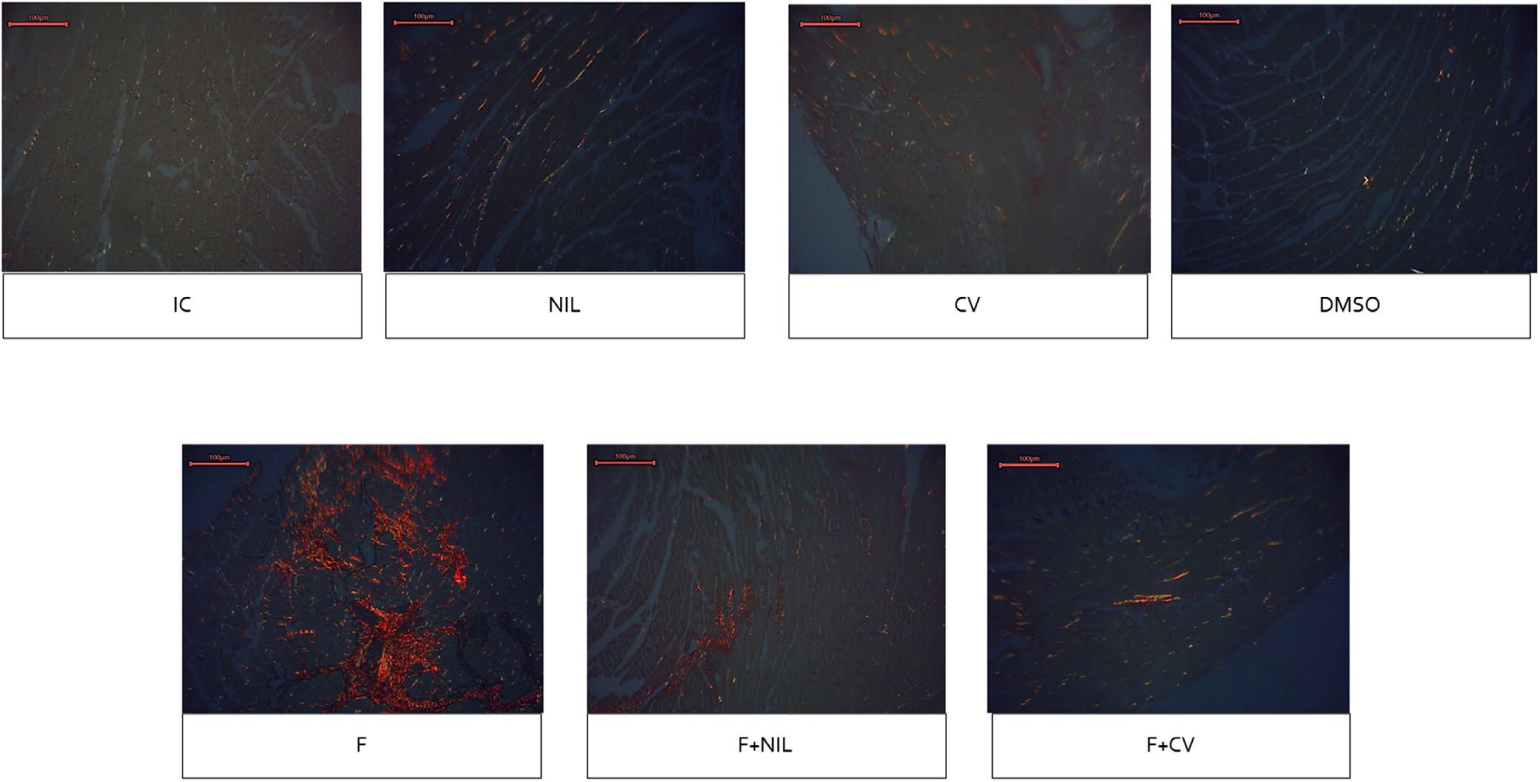
Cardiac histopathological slides under Picrosirius Red staining observed under polarized light, 10× magnification. In the IC, NIL, CV, and DMSO groups, collagen fibers exhibit weak yellow‐green birefringence, consistent with type III collagen and a normal extracellular matrix. In the F group, there is intense orange‐red birefringence, characteristic of type I collagen fibers, densely and irregularly distributed, confirming advanced fibrosis and mature collagen deposition. In F+NIL, fibers display mild and discontinuous birefringence, suggesting predominantly type III collagen and immature fibers, reflecting active regression of fibrosis. In F+CV, birefringence is faint and homogeneous, without dense aggregates, and thin yellow‐green fibers predominate, strongly suggesting a substantial reduction in collagen maturation and preservation of myocardial structural integrity. These pictures show the same fields as in Figure 10. Scale bar = 100 μm. Number of animals per group: IC (*n* = 10), NIL (*n* = 7), CV (*n* = 10), DMSO (*n* = 10), F (*n* = 5), F+NIL (*n* = 5), and F+CV (*n* = 8).

**FIGURE 12:**
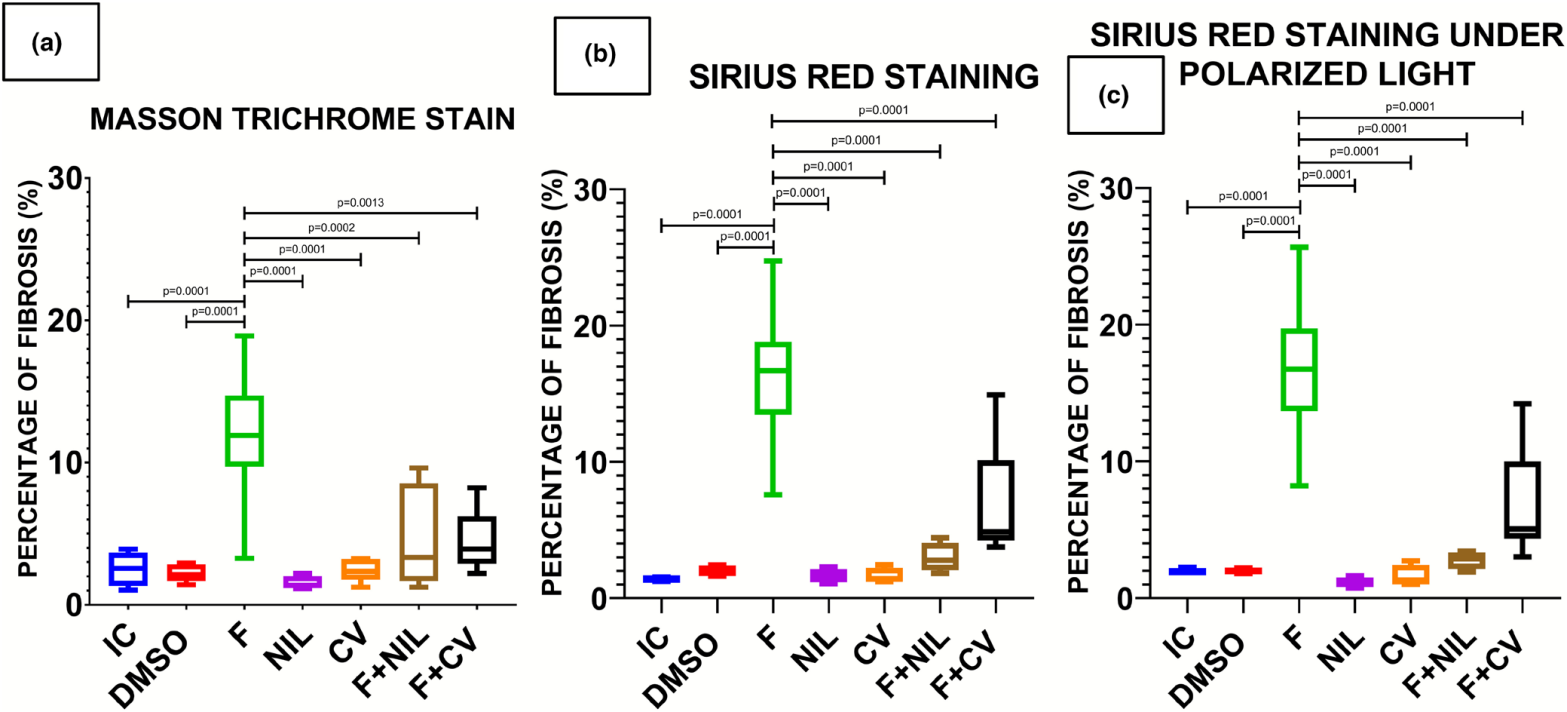
Percentage of myocardial fibrosis. (a) Masson's trichrome staining, (b) Picrosirius Red staining, and (c) Picrosirius Red under polarized light. All three staining methods revealed a significant increase in the percentage of fibrosis in the F group compared with the remaining experimental groups (*p* < 0.05). The F+NIL and F+CV groups showed values comparable to the control group, demonstrating a reduction in the fibrotic process. Results are expressed as mean ± SD; statistical analysis was performed using one‐way ANOVA followed by Student's *t*‐test. Number of animals per group: IC (*n* = 10), NIL (*n* = 7), CV (*n* = 10), DMSO (*n* = 10), F (*n* = 5), F+NIL (*n* = 5), and F+CV (*n* = 8).

Cardiac histopathology is shown in Figures 8, 9, 10, 11. IC, NIL, DMSO, and CV groups showed myocardium with preserved architecture, aligned and compact cardiomyocytes, minimal connective interstitium, and absence of fibrosis. These findings were consistent across H‐E, Masson's trichrome, and picrosirius red stains, in which collagen appeared scarce, thin, and uniformly distributed, with yellow‐green birefringence under polarized light, characteristic of type III thin fibers. In contrast, the F group exhibited marked tissue disorganization, cardiomyocyte hypertrophy, diffuse interstitial expansion, and abundant type I collagen deposition, evidenced by intense blue staining with Masson, and bright red with orange birefringence under polarized light with picrosirius red, indicative of advanced interstitial and perivascular fibrosis. The F+NIL group showed improved structural organization, reduced interstitial thickness, and decreased collagen content, with a predominance of type III collagen thin fibers, suggesting partial regression of fibrosis. The F+CV group exhibited an almost normal morphological pattern, with compact muscle fascicles, minimal collagen deposition, and faint yellow‐green birefringence, indicating substantial fibrotic regression. Figure 12, summarizes the fibrosis percentages derived from Masson's trichrome, picrosirius red, and picrosirius red under polarized light, demonstrating a significant increase in fibrosis in the F group compared to all other groups, while F+NIL and F+CV maintained values comparable to controls, confirming attenuation of cardiac fibrotic remodeling with both interventions.

## 4 DISCUSSION

Abdominal aortic stenosis (AAC) is a widely used experimental model to induce cardiac fibrosis in laboratory animals; it has the capacity to reproduce the structural and functional changes observed in chronic human hypertensive heart disease (Ajith Kumar et al., 2014). Analogous to hypertensive cardiopathy, AAC first elicits perivascular fibrosis, which subsequently progresses to diffuse interstitial fibrosis, generating electrical heterogeneity and a higher susceptibility to cardiac arrhythmias. These changes are also associated with reduced exercise capacity and lower survival, mirroring functional alterations reported in patients with hypertrophy and myocardial remodeling due to chronic pressure overload (Weber, 2004).

At the cardiac level, arginine vasopressin (AVP) exerts a direct profibrotic effect, primarily via V1a receptor activation, which stimulates PKC and subsequently the NF‐κB signaling pathway, promoting TGF‐β expression (Fan et al., 2007). Activation of this cascade triggers cell‐proliferative signals and a direct myogenic effect on myofibroblasts, favoring type I and III collagen synthesis and fibrotic myocardial remodeling. This phenomenon has been shown to be V1a‐specific in prior experiments (Yang et al., 2003). In hepatic models, V1a receptor blockade halts fibrosis progression and even induces fibrosis regression (Navarro‐Gonzalez et al., 2023). On this basis, our central hypothesis posits that AVP deficiency, or its blockade, directly reduces the cardiac fibrotic process and improves clinical, biochemical, and histological outcomes, whether via NIL surgery or CV treatment.

Survival curves showed a marked reduction in the F group survivance, likely attributable to progressive heart failure secondary to AAC‐induced myocardial fibrosis; notably, postmortem examinations revealed findings consistent with heart failure, including fluid overload, myocardial fibrosis, and myocardial necrosis. In contrast, F+CV exhibited a significant survival benefit, suggesting that V1a receptor blockade not only confers a direct antifibrotic effect but also improves overall survival by mitigating the hemodynamic consequences of fibrosis. Conversely, the notable reduction in survival in F+NIL can be explained by surgical trauma associated with NIL, as mortality clustered in the peri‐ and immediate postoperative period. We hypothesize this reflects pre‐existing comorbidities; at the time of NIL, these animals already exhibited clinical signs of advanced heart failure, increasing postoperative vulnerability and secondary to the severe hypotensive immediate effect mediated by the acute AVP deficiency.

Although total heart weight did not differ significantly among groups, marked biventricular hypertrophy was evident in F, consistent with classic morphological features of hypertensive cardiopathy (Figure 4). Importantly, AVP deficiency or its blockade in F+NIL and F+CV groups was associated with significant regression of ventricular hypertrophy, indicating a cardioprotective effect. This may represent an indirect manifestation of fibrosis reduction, given that cardiac hypertrophy is a biomechanical compensatory response to chronic pressure overload that, if sustained, progresses to structural remodeling, interstitial fibrosis, and ventricular dysfunction (Nwabuo & Vasan, 2020).

Tail‐cuff systolic blood pressure decreased significantly in AAC groups (F, F+NIL, and F+CV) relative to remaining groups. This was expected since ligation at a lower level of the renal arteries reduces blood flow to the caudal artery and thus reduces caudal pressure (Ku et al., 2016). Notably, in the third measurement, the NIL and CV groups—despite not undergoing AAC—also showed significant caudal pressure reductions. This aligns with prior observations from our laboratory indicating that AVP deficiency is associated with lower mean arterial pressure via a mechanism independent of RAAC (Villanueva et al., 2015). In contrast, carotid mean arterial pressure was significantly increased in F, consistent with prior reports describing this expected effect of the AAC model due to elevated afterload and proximal hemodynamic remodeling, both through direct hemodynamic effects and chronic RAAC activation (Kim et al., 2021). These findings confirm that the heart failure phenotype and myocardial fibrosis in this model are secondary to chronic hypertensive processes. Conversely, the F+NIL and F+CV groups exhibited marked reductions in carotid MAP, reaching physiological levels. Beyond the direct antifibrotic effects on cardiac myofibroblasts, these findings highlight that vasodilation (via V1a receptor blockade) and, particularly, renal volume depletion (via V2 receptor inhibition) synergistically contribute to an improved hemodynamic profile. This dual mechanism—attenuation of myocardial fibrosis together with afterload and preload reduction—appears to underlie the overall therapeutic benefit of AVP deficiency or pharmacological blockade in hypertensive heart failure.

In resting ECG analyses, the F group exhibited a high relative risk (RR) of arrhythmias, reaching 4.501 at week 9. The most frequent abnormalities were right/left bundle branch blocks and peaked Q waves, classic indirect parameters indicative of myocardial fibrosis (Nguyen et al., 2014). The pathogenesis of these arrhythmias in the context of fibrosis involves gap junction uncoupling and desynchronization from physiological pacemakers, favoring ectopic foci, reentry circuits, and electrical alternans. These phenomena result from disruption of normal conduction tissue by interstitial collagen infiltration, which alters impulse propagation and creates an arrhythmogenic substrate, as described in prior fibrosis models (Verheule & Schotten, 2021). In F+NIL and F+CV, RR declined from 4.501 to 1.385 and from 4.909 to 2.929, respectively, suggesting that both AVP deficiency (NIL induced) and its pharmacological blockade (CV induced) were associated with reduced myocardial fibrosis deposition, yielding a more stable conduction system with fewer abnormalities than the untreated group. This same pattern was reproduced in post‐exercise ECGs, reinforcing the interpretation: during exercise, myocardial excitability increases, as does the likelihood of provoking arrhythmias—especially mild or subclinical forms that may be silent at rest—due to catecholamine surge and sympathetic stimulation (Cheung et al., 2016).

A well‐recognized early clinical manifestation of hypertensive cardiopathy is the reduced exercise capacity, secondary to impaired myocardial contractility under hemodynamic stress induced by fibrosis. The AAC model reproduces this phenomenon: pressure overload leads to hypertrophy, ventricular stiffness, and progressive functional decline (Kim et al., 2021). Then, our findings support this view since at week 5, AAC‐exposed rats showed significantly reduced exercise capacity. By week 11, this worse in F, whereas F+CV and F+NIL demonstrated substantial recovery to near‐physiological exercise performance. The onset of diabetes insipidus in F+CV and F+NIL groups induced a profound volume depletion, thereby reducing cardiac preload; this hemodynamic adjustment, together with the potential direct antifibrotic effect of AVP deficiency, likely translated into the improved exercise performance observed. This is particularly relevant, showing that AVP blockade/deficiency not only yields favorable antifibrotic and electrical effects, but also improves functional capacity, reflecting integral cardiovascular recovery and a better “quality of life” on treated animals.

Histopathologically, cardiac fibrosis is identified by interstitial and perivascular collagen accumulation, whose features vary by stain. With H‐E, separation between cardiomyocytes and loss of fascicular organization is observed, with slightly eosinophilic collagen fibers. In Masson's trichomic stain, the collagen fibers acquire an intense blue coloration, delineating replacement of muscular tissue for fibrotic connective tissue. Picrosirius red stains collagen with bright red color, and under polarized light distinguishes type I (orange) from type III (greenish) collagen, enabling a more specific assessment of fiber maturity and remodeling grade (Frangogiannis, 2021; Junqueira et al., 1979). In our study, both histopathology and fibrosis quantification showed that F exhibited features of an active fibrotic process—predominantly interstitial and perivascular—was observed across all stains, thus confirming the success of the AAC model. In contrast, F+CV and F+NIL groups displayed a pattern of residual fibrosis, characterized by significantly reduced collagen accumulation and a shift from type I (pathological) toward type III (physiological remodeling) collagen. These findings confirm that both AVP deficiency and pharmacological AVP blockade promoted reduction and regression of fibrosis in AAC‐subjected animals, demonstrating a structurally verifiable antifibrotic effect. Future studies will include the assessment of profibrogenic and antifibrogenic gene expression to further substantiate these findings; however, such molecular analyses were not performed in the present study due to budgetary limitations and are planned for subsequent experimental phases.

## 5 LIMITATIONS

This study has several limitations that should be acknowledged. First, only male Wistar rats were included; therefore, potential sex‐specific differences in the cardiac fibrotic response to AVP deficiency or blockade were not addressed. Future studies incorporating female animals are necessary to determine whether these findings are influenced by sex‐related hormonal or molecular factors. Second, circulating AVP levels were not directly measured due to budgetary constraints. However, previous work from our group has consistently demonstrated a marked reduction in AVP levels following neurointermediate pituitary lobectomy (Organista‐Esparza et al., 2016). In addition, all NIL animals underwent postmortem autopsy at the end of the experimental period to confirm complete neurointermediate pituitary resection, and animals with incomplete lobectomy were excluded from the analysis. Finally, although the present study focused on functional, histological, and morphometric outcomes, the assessment of specific pro‐fibrogenic and anti‐fibrogenic molecular pathways (e.g., through zymography and gene expression analyses) was not performed. These analyses will be addressed in the next phase of the project, as their inclusion was not feasible in the current study due to financial limitations.

## 6 CONCLUSIONS

The results of the present study demonstrate that both AVP deficiency induced by NIL and its pharmacological blockade with CV effectively decrease and reverse cardiac fibrosis induced by hypertensive cardiopathy (abdominal aortic stenosis model). This effect was confirmed through histological evaluation, clinical variables (blood pressure, resting electrocardiogram, exercise test, and functional exercise capacity).

These findings suggest that the underlying mechanism involves both a direct antifibrotic action on cardiac myofibroblasts, by suppressing V1a receptor activation and promoting an antifibrotic cellular phenotype, and an indirect hemodynamic effect mediated by water depletion secondary to V2 receptor blockade at the renal level. Together, these effects translate into structural and functional improvement of the myocardium.

Future studies are warranted to further elucidate the molecular mechanism of this effect by immunohistochemistry (α‐SMA, TGF‐β, SMAD, collagen type I and III) and RT‐PCR (MMP2, MMP3, and TIMP1), as well as to compare the efficacy of conivaptan against conventional heart failure therapies (diuretics, angiotensin II receptor antagonists, and angiotensin‐converting enzyme inhibitors).

Collectively, our findings propose that vasopressin V1a/V2 receptor blockade may represent a promising alternative therapeutic strategy for the treatment of myocardial fibrosis and hypertensive heart disease, although additional studies are required to confirm its safety, efficacy, and clinical applicability.

## AUTHOR CONTRIBUTIONS

Limón‐Mendoza and Quintanar‐Stephano were responsible for the experimental design, supervision, data interpretation, statistical analysis, manuscript writing, and final approval. Alamilla‐Rasso, González‐Nuñez, Becerra‐Mendez, and García‐Álvarez conducted the experimental procedures, while Tinajero‐Vidales and Tinajero‐Ruelas were responsible for the histopathological techniques, the histopathological and statistical analyses of the results obtained from these histological sections.

## FUNDING INFORMATION

This study was funded by the PIFF 25‐1 Grant from the Universidad Autónoma de Aguascalientes, awarded to Dr. Andrés Quintanar‐Stephano.

## CONFLICT OF INTEREST STATEMENT

The authors declare no conflicts of interest.

## ETHICS STATEMENT

The experimental protocol was approved by the Institutional Bioethics Commite of the Universidad Autonoma de Aguascalientes (approval ID CEADI‐UAA/02/2024).

## Data Availability

Data will be made available upon a reasonable request via the corresponding author.
